# Extended-Aperture Shape Measurements Using Spatially Partially Coherent Illumination (ExASPICE)

**DOI:** 10.3390/s24103072

**Published:** 2024-05-12

**Authors:** Mostafa Agour, Claas Falldorf, Ralf B. Bergmann

**Affiliations:** 1 BIAS—Bremer Institut für angewandte Strahltechnik, 28359 Bremen, Germany; falldorf@bias.de (C.F.); bergmann@bias.de (R.B.B.); 2 Physics Department, Faculty of Science, Aswan University, Aswan 81528, Egypt; 3 MAPEX Center for Materials and Processes and Faculty of Physics and Electrical Engineering, University of Bremen, 28359 Bremen, Germany

**Keywords:** optical metrology, shape measurements, partially coherent illumination, depth discrimination

## Abstract

We have recently demonstrated that the 3D shape of micro-parts can be measured using LED illumination based on speckle contrast evaluation in the recently developed SPICE profilometry (shape measurements based on imaging with spatially partially coherent illumination). The main advantage of SPICE is its improved robustness and measurement speed compared to confocal or white light interferometry. The limited spatial coherence of the LED illumination is used for depth discrimination. An electrically tunable lens in a 4f-configuration is used for fast depth scanning without mechanically moving parts. The approach is efficient, takes less than a second to capture required images, is eye-safe and offers a depth of focus of a few millimeters. However, SPICE’s main limitation is its assumption of a small illumination aperture. Such a small illumination aperture affects the axial scan resolution, which dominates the measurement uncertainty. In this paper, we propose a novel method to overcome the aperture angle limitation of SPICE by illuminating the object from different directions with several independent LED sources. This approach reduces the full width at half maximum of the contrast envelope to one-eighth, resulting in a twofold improvement in measurement accuracy. As a proof of concept, shape measurements of various metal objects are presented.

## 1. Introduction

Accurately measuring the geometry of technical parts is crucial for improving and streamlining the manufacturing process [1]. To ensure quality during production, 3D shape profiling is essential for many applications. Micro-parts, with dimensions smaller than 1 mm, are produced in large quantities for use in industries such as automobile manufacturing [2]. These micro-parts form the building blocks of larger assemblies and thus often require 100% inspection to guarantee defect-free components for many applications [3,4]. In an industrial environment, the utilization of optical metrology necessitates not only accurate measurement of product geometry but also short measurement times and resistance to mechanical distortions [5], especially vibrations. Moreover, when analyzing microscopic objects, the measurement technique should additionally offer an enhanced depth of focus [6].

To address the above requirements, we have recently proposed a method based on a common-path 4f-imaging system using LED illumination [4]. In analogy to phase retrieval [7] and shear interferometry [8], the adoption of a common-path configuration enhances the robustness of the setup against vibrations, distinguishing it from interferometric-based methods [9] and white light interferometry (WLI) [10]. To facilitate fast measurements, we have incorporated an electrically tunable lens (ETL), which is adjusted in the common Fourier plane of the 4f-imaging system. The ability to change the effective focal length of the ETL allows for rapid axial scanning of the object, a notable advantage over WLI [10], without the need for mechanically moving parts. In this configuration, the complex transmission of the lens modulates the light generated in the common Fourier plane with the transfer function of propagation [11], which is analogous to the use of a spatial light modulator in phase retrieval [12,13]. The 4f-imaging system is essentially a spatial filter, as shown by the analysis of its transfer function, limiting the coherent modes passing its aperture [14]. Consequently, this method uses spatially partially coherent illumination to encode depth information as an intensity contrast at the output of the imaging system [15]. We have termed this distinctive approach of shape measurements based on imaging with spatially partially coherent illumination as *SPICE profilometry*.

A similar technique, known as focus variation microscopy (FVM) [16], reconstructs the 3D shape and surface topography of a test object [17] by identifying the optimal focus position within a captured set of images acquired during axial or vertical scanning. The best focus is determined using a focus measure or metric based on the standard deviation or contrast of a small window around each pixel in the captured images. In contrast to FVM, which relies on a large numerical aperture of the imaging system, SPICE is entirely based on a large numerical aperture of incoherent illumination. However, previous implementations of SPICE required the illumination aperture to be small compared to the numerical aperture of the imaging system, limiting the axial scan resolution and thus the achievable measurement uncertainty. This limitation prevents SPICE from tapping its full potential.

In this paper, we investigate a method to overcome the limitation of SPICE’s limited axial resolution by combining measurements with multiple illumination directions, which is achieved by illuminating the object with several independent light sources, thus reducing the full width at half maximum of the contrast envelope. We will refer to the proposed method as extended-aperture SPICE or *ExASPICE*. The new approach, the impact of the illumination scheme, and the experimental verification using a technical object are discussed in detail in the present paper. In addition, focus measures and limitations are defined, as well as methods to improve the measurement in terms of contrast enhancement and reconstruction, e.g., pre-processing the acquired image set by enhancing its signal-to-noise ratio. Finally, we show a comparison with established shape measurement methods to demonstrate the validity of ExASPICE.

## 2. Form Measurements Based on Intensity Contrast

In our previous work [4], we demonstrated that illumination using an LED with its limited spatial coherence in conjunction with a 4f-imaging system enables depth discrimination. It is important to note that this method requires the measured object to possess a rough surface.

To understand the principle of SPICE, we have to consider the LED light source as an extended light source composed of *N* independent elementary sources. Each elementary source will create a fully developed speckle field across the image plane. All of the speckle fields will add incoherently; e.g., their intensities will add because the elementary sources are mutually independent. For those parts of the object that are in focus, all of the speckle fields coincide, giving rise to a full contrast speckle pattern. For object parts outside the focal plane, the speckle fields will shift depending on the angle of the corresponding elementary source with the observation direction, and hence the speckle fields will rapidly wash out. The maximum shift Δx(z) in the individual (elementary) speckle fields concerning the out-of-focus distance *z* can be determined by [15]

(1) $$\Delta x\left(z\right)=z\cdot \theta_{ill}=z\frac{r_{s}}{2z_{ill}}\;.$$

Here, $\theta_{ill}$ is the angular diameter of the illumination, which determines the numerical aperture of the illumination, $z_{ill}$ denotes the distance between the light source and the surface under test, and $r_{s}$ is the source diameter.

With a radius of the point spread function of the 4f-imaging system of δx=1.22λf/D, with *D* denoting the diameter of an aperture located at the common Fourier plane, the contrast disappears at Δx>δx. According to the heuristic contrast model we developed for phase retrieval in [15], the contrast *K* can be written as

(2) $$K\left(z\right)=\left\{\begin{array}{cc} 1-{\left(\frac{\Delta x(z)}{\delta x}\right)}^{2}, & \mathrm{for}\;\Delta x\leq \delta x\\ 0\;, & \mathrm{otherwise}. \end{array}\right.$$

According to Equation (1), for out-of-focus object points, Δx(z) increases and thus K(z) decreases. The width $W_{z}$ of the contrast curve is defined by setting Δx(z)≤δx and substituting the definitions of Δx(z) and δx, leading to the following expression:

(3) $$W_{z}\approx \frac{\delta x}{{\mathrm{NA}}_{ill}}\;.$$

With this principle in mind, an important requirement is that the individual speckle patterns have the same structure and only differ by their average propagation direction. Because of the limited numerical aperture of the imaging system (e.g., due to the diameter *D*), this can only be guaranteed for small variations in the illumination angle $\theta_{ill}$. If this condition is not met, the elementary speckle fields will decorrelate and hence also wash out for objects parts which are in focus.

## 3. Increasing the Illumination Aperture

**Figure 1:**
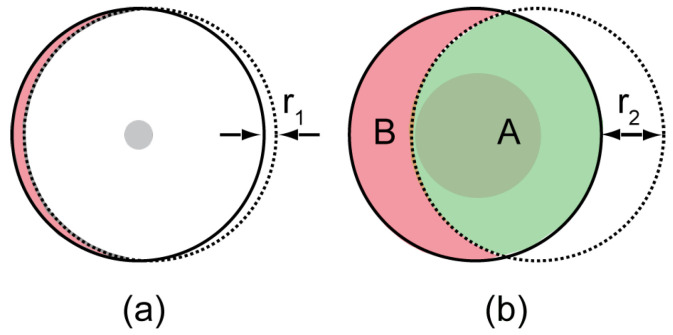
Enlargement of the illumination aperture: The solid circles in both figures schematically represent the Fourier plane of the imaging system under normal incident illumination. When the illumination direction changes, the wavefield in the aperture area is shifted, as indicated by the dashed circles. Here, the horizontal shift corresponds to a maximum horizontal tilt of a plane wave illumination $k_{x}$. According to Equation (4), the numerical aperture of the illumination ${\mathrm{NA}}_{ill}$ is proportional to $k_{x}$. (**a**) Situation where Equation (1) holds for only a small variation in the illumination direction corresponding to a shift at the Fourier plane with a magnitude of $r_{1}$. The effective numerical aperture of the illumination is small, as indicated by the small gray disc of radius $r_{1}$. This small shift introduces a new wavefield within the red area that minimally affects the speckle pattern formed by the normal illumination direction. (**b**) Large variation in the illumination angle compared to (**a**), resulting from a larger spacial frequency $k_{x}$ and thus a larger numerical aperture indicated by the larger gray disc of radius $r_{2}$. Here, the aperture is divided into two regions indicated by the letters A (green area) and B (red area). A contains wavefields that have correlated speckles; i.e., the generated speckle fields have the same features but are shifted. However, B contains uncorrelated speckles, i.e., speckle fields with different features affecting the overall speckle contrast in the camera plane.

In SPICE, the motivation behind the assumption of a small illumination angle is that the speckle field generated in the image plane is assumed to be approximately identical and independent of the illumination direction, see Figure 1a for more details. In this paper, however, we aim to experimentally investigate whether this assumption can be relaxed, potentially improving both the sensitivity and the accuracy of the proposed approach.

Predictably, increasing the angle of illumination means effectively changing the direction of illumination. This will simply result in different directions of propagation, causing a lateral shift across the common Fourier plane of the imaging system in which an aperture is located, as shown in Figure 1b. Such large variations in the illumination direction will violate the small illumination angle assumption used for Equation (1).

Here, we plan, according to Equation (3), to reduce $W_{z}$ by increasing the illumination aperture. Thus, we investigate the scenario where the variations in illumination directions are much larger, so that Equation (1) no longer holds. This situation is shown in Figure 1b. Here, the dashed circles indicate the shift in the wave field in the aperture plane due to a change in illumination direction. However, this time, the shift is much larger than in Figure 1a, which shows a small numerical aperture. In Figure 1b, one can divide the aperture into different regions, as indicated by the letters A and B. In region A, we find those parts of the wavefield that are present under normal illumination but shifted. In region B, we have new wavefield information outside the aperture in the case of normal illumination. Light coming from either region will behave very differently around the image plane. Light coming from A will behave according to Equation (1) because it adheres to the underlying assumption that the wavefield is only shifted in the Fourier domain. In particular, across the image plane, where light from A will produce the same speckle field regardless of the direction of illumination, light from B will produce a different speckle field for each illumination direction. This leads to uncorrelated speckle fields and hence, the overall speckle contrast is reduced. Taking into account that

(4) $${\mathrm{NA}}_{ill}=k_{x}/k\;\mathrm{and}\;{\mathrm{NA}}_{img}=k_{obj}/k\;,$$

with the transferred object frequencies $k_{obj}$ allowed by the objective, Equation (3) can be rewritten as

(5) $$W_{z}=0.61\lambda \frac{1}{{\mathrm{NA}}_{ill}\cdot {\mathrm{NA}}_{img}}\;,$$

with the numerical aperture of the imaging system ${\mathrm{NA}}_{img}=D/2f$. For the sake of simplicity, the explicit dependence on *k* is omitted. Please note that Equation (5) gives the trade-off between the axial resolution and ${\mathrm{NA}}_{ill}$ and ${\mathrm{NA}}_{img}$. The main objective now is to find the minimum width of the contrast envelope, $W_{min}$, which can be achieved via

(6) $$W_{z,min}=\min_{k_{x,max}}\left\{W_{z}\right\}\;,$$

where $k_{x,max}$ is the maximum allowable Fourier domain shift defined by the effective numerical aperture of the illumination. According to Equation (5), this minimum is reached when ${\mathrm{NA}}_{ill}$ and ${\mathrm{NA}}_{img}$ are are equal, so it can be rewritten as

(7) $$W_{z,min}=0.61\lambda \frac{1}{{\mathrm{NA}}^{2}}\;,$$

with $\mathrm{NA}={\mathrm{NA}}_{ill}$ = ${\mathrm{NA}}_{img}$. This value corresponds to the Rayleigh length, or in other words, the depth of focus that ensures a maximum speckle contrast. Thus, Equation (7) describes the minimum axial resolution of the ExASPICE technique.

Note that $W_{z,min}$ is also equal to the width of the focus measure envelope in FVM. Thus, the axial resolution is the same in both techniques. However, the main advantage of ExASPICE over FVM is that no surface textures or features are required in the reconstruction process. The main limitation of both ExASPICE and SPICE profilometry is that the object surface must be optically rough. This causes speckle to be generated and evaluated at the imaging plane. To overcome this, structured illumination using a rotating diffuser [18,19] can be used to investigate objects with smooth surfaces. In the following, two measurement scenarios will be discussed, one for the extreme case where ${\mathrm{NA}}_{ill}>{\mathrm{NA}}_{img}$, indicating no speckle contrast, and the second for ${\mathrm{NA}}_{ill}={\mathrm{NA}}_{img}$.

## 4. Experimental Results

**Figure 2:**
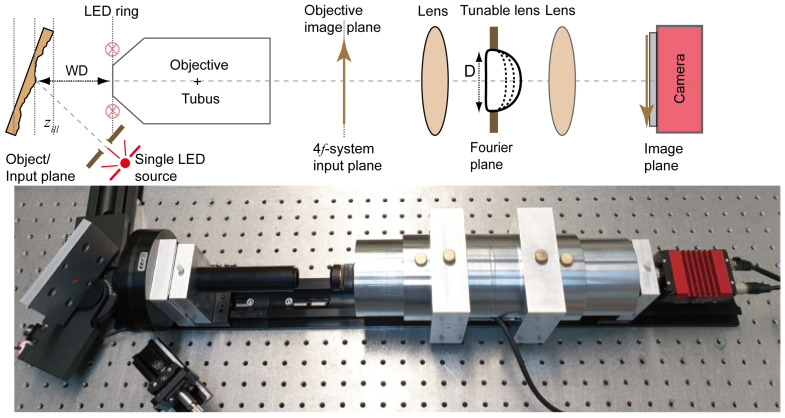
Experimental setup of the 3D profiler. A plate with an optically rough surface is used as a test object and is illuminated by light emitted from an LED array with $z_{ill}$ referring to the distance between the illumination and the test object. The object is tilted by an angle of $45^{\circ }$ with respect to the input plane of the imaging system and is placed at the object plane of a 10× microscope objective with a numerical aperture of 0.21 and a working distance (WD) of 51 mm. The ETL used here is the EL-10-30-C-VIS-LD-MV with an aperture D of 10 mm and is supplied by Optotune Switzerland. A camera sensor (Prosilica GT 2750 supplied by Allied Vision Technologies GmbH, Osnabrück, Germany) is positioned at the camera plane.

Figure 2 shows the setup used to obtain a proof of concept by investigating the contrast variation across a tilted metallic plate. The metallic plate is fixed on a rotation stage (which has a precision of ≈15 μrad) and tilted by an angle of $45^{\circ }$ with respect to the input plane of the imaging system. The plate is positioned at the working distance (WD) of a microscope objective, which has a magnification (*M*) of 10× and a numerical aperture of 0.21. Therefore, the relationship between the propagation distance at the object plane Δz and the image plane *z* is given by $z=M^{2}\Delta z$. The illumination of the LED ring and the single LED used in this setup has an average wavelength of 630 nm and a temporal coherence length of approximately $l_{coh}\approx 11\;\mu$m. The single LED is coupled to a fiber with a diameter of $r_{s}=400\;\mu$m. The end of the fiber is fixed to a collimator with a focal length of $f_{c}=6.17$ mm, producing a beam diameter of approximately two millimeters. Thus, the angular diameter of the source is $\theta_{ill}=0.032$. Based on these parameters, the spatial coherence length is found to be $r_{coh}=11.85\;\mu$m. The optical setup follows a 4f configuration, consisting of a pair of identical lenses with a focal length of 105 mm and an electrically tunable lens (ETL). The ETL has a band-limiting aperture with a diameter of 10 mm, which is adjusted at the common Fourier plane. As a result, the imaging numerical aperture is ${\mathrm{NA}}_{img}=0.048$, the radius of the point spread function is δx=8.07μm and for the single LED case $W_{z}=167.5\;\mu$m. The propagation in this setup is achieved by electrically adjusting the focal length (FL) of the ETL, which is capable of switching in less than 2.5 ms. The camera sensor has a pixel pitch of 4.54μm and a resolution of 2750×2200 pixels.

### 4.1. Ring LED Illumination

**Figure 3:**
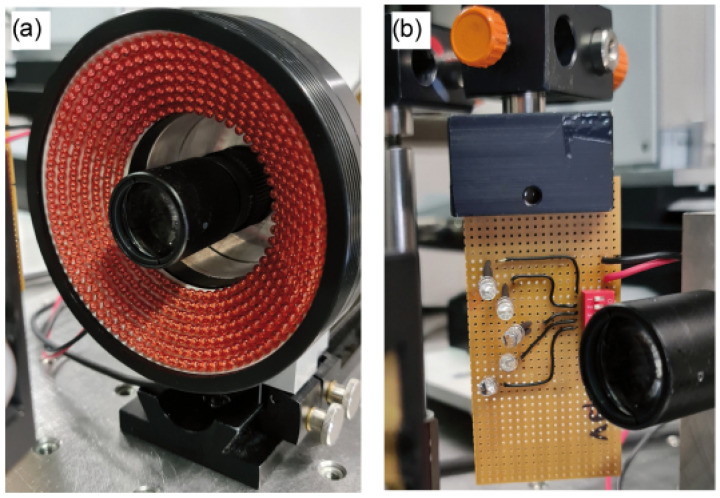
LED sources used to illuminate the test object: (**a**) represents the LED ring light source (model: CCS-LDRS2 series, supplied by Stemmer-Imaging AG). The ring light characteristics and datasheet can be found in [21]. (**b**) A home-made LED source consisting of five red LEDs mounted on a circular arc, which can be switched on manually in sequence or simultaneously.

Here, we have assumed that ${\mathrm{NA}}_{ill}>{\mathrm{NA}}_{img}$, realized by illuminating the test object with the ring illumination. As shown in Figure 3a, the ring illumination provides direct light from red LEDs mounted at an angle to the intersection of the test object and the optical axis. Note that this task was briefly presented in our recently published SPIE [20] paper. In this situation, we assumed that the contrast of the speckle at the image plane would vanish due to the contribution of shifted and uncorrelated speckle fields, as discussed in Section 3.

To prove this, a metal plate is placed so that its center is at the WD of the objective, according to the camera’s field of view. Consequently, $F_{L}$ is electrically tuned so that the center of the object is in focus and shows the highest speckle contrast. The metal plate is positioned on a rotary stage and then tilted to simulate a three-dimensional object. The plate is tilted at an angle of $15^{\circ }$ degrees (the rotation stage has a precision accuracy of about ≈15 μrad) relative to the optical axis. In terms of the surface roughness of the plate, a confocal roughness measurement using the Keyence VX-3000 gives a rough surface with $R_{a}=4.053\;\mu$m and $R_{z}=14.998\;\mu$m. This gives a good optically rough surface that produces a fully developed speckle under coherent illumination [22]. The plate is then illuminated with partially spatially coherent light emitted from either a single LED or an LED ring. Considering the camera’s resolution, the pixel size and the magnification of the microscope objective, the part of the plate projected onto the camera sensor has a width of 2200μm. Using the geometric properties of the plate, including the tilt angle and the projected width of the object, the examined height difference of the scanned object is determined to be h=342.16μm with an accuracy of approximately ±0.02 μm, resulting from the precision of the rotary stage.

**Figure 4:**
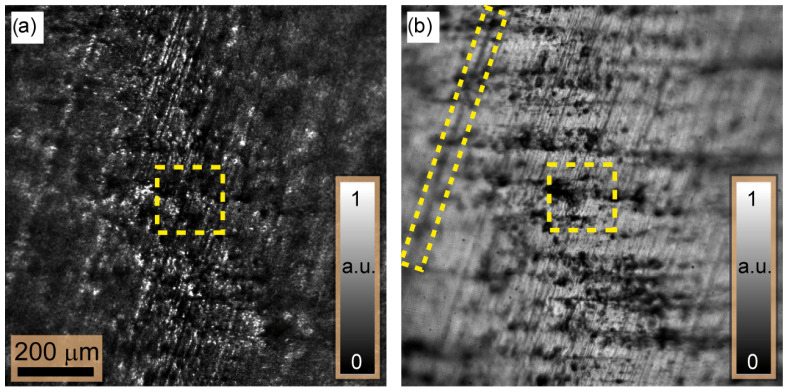
Two examples of intensity images captured for the tilted metallic plate when $F_{L}=690$ mm and using (**a**) a single LED light source and (**b**) the LED ring light. The images indicate the variation in the contrast envelopes over the transverse axes of the images. The yellow boxes show where the focus measure plotted in Figure 5 is measured. The rectangular yellow box in (**b**) highlights a scratch mark made by a scriber which leaves a deeper and wider scratch than other unmarked surface scratches and is used for comparison purposes.

Figure 4 illustrates two examples of captured images obtained at the image plane using the same tunable focal lengths of $F_{L}=690$ mm, corresponding to a propagation distance of 147μm at the object plane. These images exhibit a limited depth of focus, meaning that high-contrast intensity distributions are only guaranteed for object points situated within the input plane of the imaging system. However, for object points that are out of focus, the contrast decreases and eventually reaches its minimum, as predicted. In addition, areas with dents and peaks are difficult to detect in both images due to the lack of intensity diffracted towards the camera from these areas and appear dark. Figure 4a is taken using a single LED point source, showing the expected speckle field at the in-focus area. In Figure 4b, the same situation is repeated but using the LED array illumination where all LEDs are switched on simultaneously. Unfortunately, no speckles were observed in either the in-focus or out-of-focus areas.

**Figure 5:**
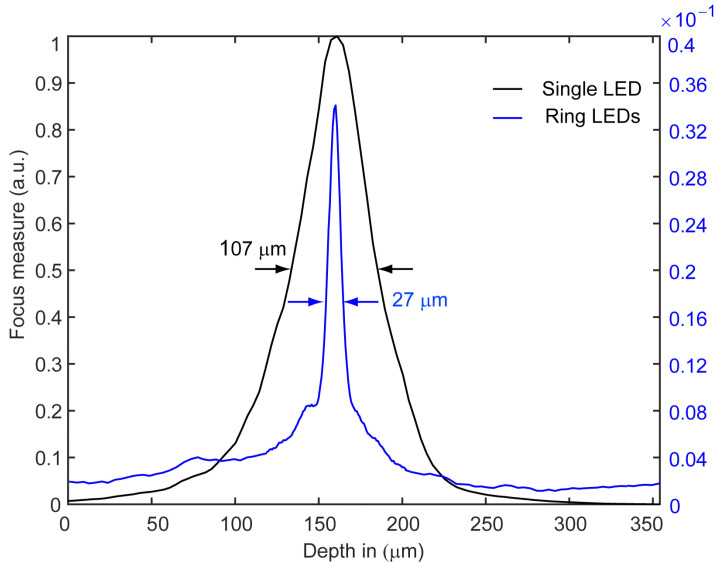
Contrast envelopes of the Michelson contrast which are used as a focus measure to define the depth within the scanning process. The two contrast envelopes are calculated using 100 captured images and for a window of 64×64 pixels defined by the yellow boxes shown in Figure 4.

By capturing a total of 100 images to scan the entire depth of the object, the widths of the contrast envelope, specifically the full widths at half maximum, are 107μm and 27μm for the single LED and ring illumination, respectively. These results are obtained for a window of 64×64 pixels at the center of the captured images and shown in Figure 5. This represents a fourfold reduction in the full width at half maximum of the contrast envelope.

The contrast in the observed ring illumination images is considerably low. Nevertheless, the maximum can be accurately determined when the image is in focus. It should be noted that the detection of the peak of the focus envelope involves polynomial curve fitting to ensure accurate peak detection [23]. Specifically, for single LED and ring illumination, the depths corresponding to the maximum contrast within the same window are 171.74μm and 175.31μm, respectively. This implies a difference of around 3.5μm, which potentially results from the low contrast. This deprives the proposed SPICE profilometry of its advantage of encoding depth information as a function of speckle contrast compared to the focus variation method, which uses surface features such as scratches or dents. However, the fast and non-mechanical depth scanning method distinguishes the extended aperture SPICE from the focus variation technique.

These findings suggest the possible requirement of an alternative focus measure. Therefore, a focus measure that uses the Gaussian derivative (GDER) [24], a widely used technique in focus variation methods [23], is employed to reconstruct the tilted plate’s 3D shape.

**Figure 6:**
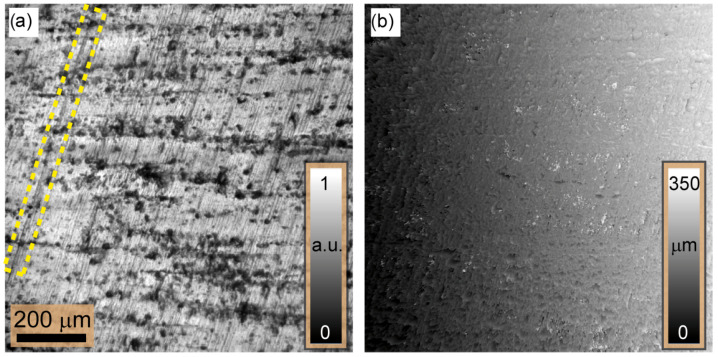
Illustration of the reconstruction of a titled metallic plate. (**a**) In-focus image of the plate surface. (**b**) Three-dimensional shape, including the surface tilt. The rectangular box denoted by a hatched line in (**a**) highlights the scratch originating from a scriber which is not visible in (**b**).

By moving an 8×8 pixel window one pixel at a time across the complete set of captured images, we reconstruct an intensity-focused image and the 3D shape of the tilted metal plate. Figure 6 displays the reconstructed results visually. This iterative process involves examining the intensity distribution within each window systematically, thereby mapping variations in GDER focus measure across the images. The reconstructed in-focus intensity image, Figure 6a, offers valuable insights into the most suitable focus position for each region, thereby enhancing comprehension of the surface features of the metal plate. For example, the scratch made by the scriber and highlighted by the rectangular box in Figure 6a is clearly visible in the reconstructed in-focus image.

The 3D shape of the metallic plate including the tilt is illustrated in Figure 6b, displaying the complexities of its surface topography. A 3D depth map of 342.5μm is obtained from the ramp modulating the plate surface. This value is very consistent with the geometrical model of the tilted plate discussed above. While the application of the Gaussian derivative focus measure guarantees a comprehensive and precise reconstruction of the three-dimensional structure of the object, the scratch made by the scriber is not visible in Figure 6b.

The metal plate’s 3D shape, along with its tilt, is visible in Figure 6b, providing insight into its surface topography. The reconstructed ramp modulation generates a thorough 3D depth map of the plate’s surface, exhibiting a value of 342.5μm. This is consistent with the geometric model of the plate of the same name discussed earlier. The use of the Gaussian derivative focus measure guarantees an accurate and detailed reconstruction of the 3D object structure. The diagram presented in Figure 6b emphasizes the efficacy of the method in precisely capturing the surface features of the metal plate.

**Figure 7:**
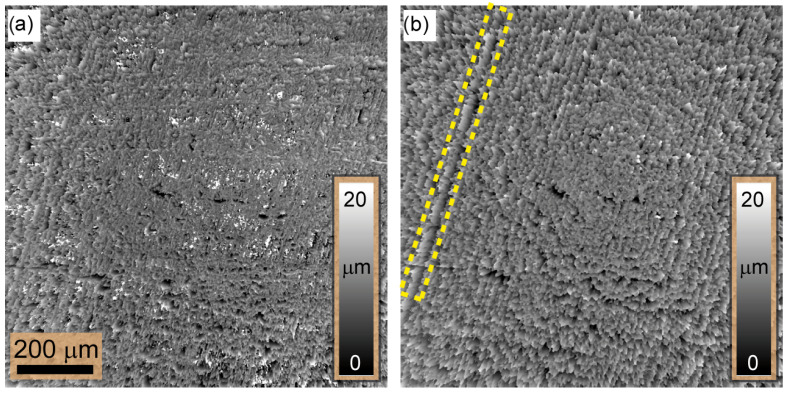
The surface of the metallic plate is displayed after compensating for the tilt: (**a**) depicts the surface topography reconstruction from the image set captured using ring LED illumination and (**b**) illustrates the reconstruction of surface topography using a single LED light source for illumination. In (**b**), more details, e.g., scriber’s scratch (yellow box), are reconstructed compared to (**a**).

Once the incline ramp was removed to correct for the tilt, the surface topography could be reconstructed. Figure 7a illustrates the outcome of this correction, which reveals that the metallic plate’s surface has a height difference between the tallest and deepest points of approximately $R_{z}=14.56\;\mu$m. This outcome closely agrees with the surface roughness obtained from Keyence confocal microscopy (VK-X3000), resulting in $R_{z}=14.998\;\mu$m. This agreement highlights the reconstruction process’s capability to capture surface irregularities and provides a quantitative assessment of surface roughness.

The surface topography constructed from the set of images taken with a single LED and obtained after tilt compensation is shown in Figure 7b. Notably, the Michelson contrast (MK) measure is employed here as the focus measure for the reconstruction. The resulting surface map renders enhanced details of the surface topography by emphasizing various scratches, particularly the scriber scratch mark, which remains unnoticed in the surface map reconstructed from the images taken under ring illumination, as depicted in Figure 7a. This is because, under a small illumination angle, i.e., numerical aperture, all generated speckle fields are correlated and can coherently superimpose. Thus, small surface variations in the range of the illumination wavelength could be encoded or observed within the contrast of the resulting speckle field. Thus, Figure 7 provides a comprehensive comparison between speckle, interference-based measurements using a single LED, and the extreme scenario where spatial coherence is distorted using ring illumination.

### 4.2. Special Illumination Configuration for ExASPICE

In this subsection, we have examined the case where ${\mathrm{NA}}_{ill}\approx {\mathrm{NA}}_{img}$. To demonstrate this process, we have repeated the measurements made for the tilted plate using a new configuration of the illumination, but using the remaining setup settings as they were. As a light source, five LEDs are arranged in an arc around the objective with a constant angular distance of $10^{\circ }$, where they will be sequentially switched on; see Figure 3b for further details. Each LED source has an average wavelength of λ=630 nm and a temporal coherence length of τ=14 nm. Moreover, each has a viewing half angle of $12^{\circ }$, which corresponds to a numerical aperture of ${\mathrm{NA}}_{ill}=0.2079$. Such illumination produces a beam diameter of 10 mm at a distance of 47 mm. Based on these parameters, the spatial coherence length is $l_{c}=13\;\mu$m. Using these geometric parameters, including those of the 4f configuration, $W_{z}=8.8\;\mu$m, where its value for a single LED source is $W_{z}=62.5\;\mu$m, obtained from Equation (3). This reduces the full width at half maximum of the contrast envelope to one eighth.

**Figure 8:**
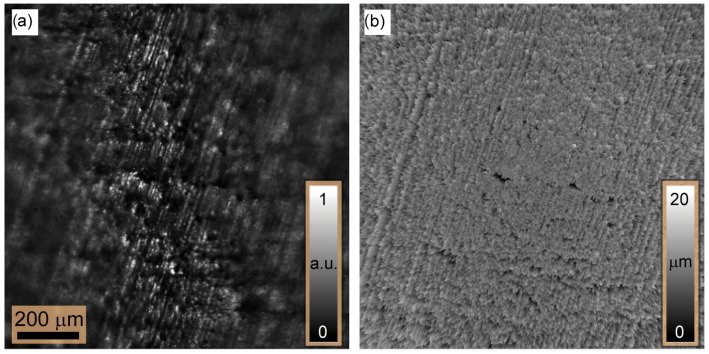
(**a**) One example of the intensity captured for the tilted metallic plate at $F_{L}=960$ mm, while the image shown in (**b**) represents the surface map of the metallic plate which is obtained after removing the incline ramp which corresponds to the applied tilt.

To determine the shape of the tilted plate, five sets of 100 intensity images are recorded in axial steps of 3.5 µm where the LEDs are switched on sequentially. Following the measurement, all (five) images at the same axial position are incoherently added to form a single intensity distribution. For comparison reasons, the intensity image resulting at $F_{L}=690$ mm is shown in Figure 8a. In contrast to the image shown in Figure 4a, one can see that outside the focus plane, the speckle vanishes, as expected. However, the speckle contrast in the focus area is comparable in both images. For a quantitative assessment, we defined MK values within a window of 32×32 pixels at the same position for the two images, which are given by 0.91 (Figure 4a) and 0.86 (Figure 8a). The small difference in MK is due to the contribution of the decorrelated speckle fields. By subjecting the measurements to the MK reconstruction approach, a 3D height map of 342.35μm is obtained and shown in Figure 8b. It agrees well with the geometrical model of the plate. From this map, the surface has an $R_{z}=14.65\;\mu$m, which is closer to the value obtained via confocal microscopy. This indicates a twofold measurement accuracy enhancement in contrast to the single LED measurement.

**Figure 9:**
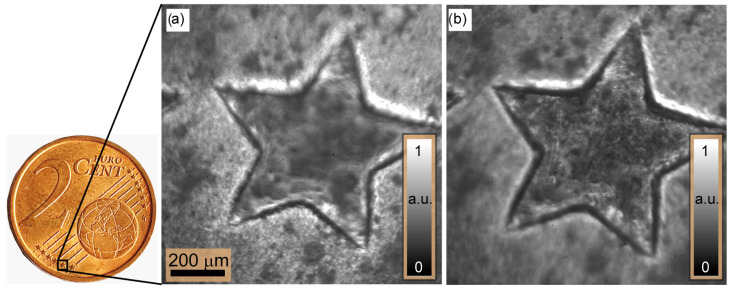
Measured intensity distributions of the star parts of a 2 cent (EUR) coin shown in the insert: intensity images captured at the image plane of the 4f-imaging system after modulating the wave field by lenses with (**a**) $F_{L}=0$ mm and (**b**) $F_{L}=958$ mm.

A second measurement example is presented to access the new proposed illumination scheme; here, the shape of a part of the 2 cent (EUR) coin is determined, see the insert in Figure 9. To achieve this, the imaged part was placed at the working distance (WD) of the microscope objective.

For shape determination, five sets of 100 intensity images are acquired with an axial step of 0.5μm. This step is chosen based on the validation analysis presented in Section 3, below $W_{z,min}$. This is achieved consequentially by switching on the LEDs. Hereafter, at each capturing axial position, all images are incoherently added in a single intensity distribution. This result will be used for contrast evaluation. Figure 9 shows two intensity distributions at two different focal lengths of $F_{L}=0$ mm and 958 mm, corresponding to a propagation distance of 0μm and 115μm in the object plane, respectively. The time required to capture each frame is 3 ms, so the entire set of images is acquired in less than one minute.

**Figure 10:**
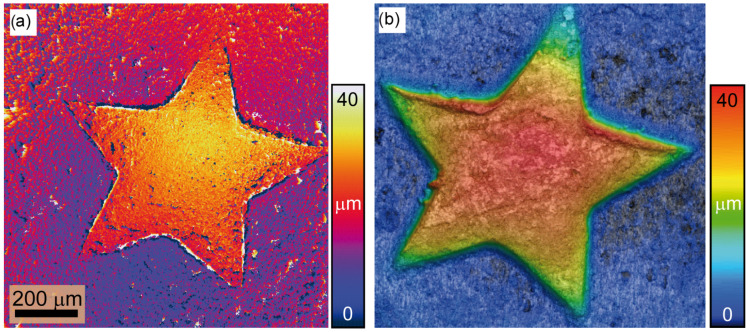
The reconstructed 3D height maps: In (**a**), the 3D map reconstructed based on the ExSPICE method is shown. In (**b**), the map was measured using Keyence scanning laser confocal microscopy using an objective of 10× which is used for comparison purposes.

The 3D form of the star using MK in a sliding window of 32×32 pixels as a measure for the contrast evaluation is reconstructed and shown in Figure 10a.

It can be seen that the surface is well reconstructed over the entire axial extent. An analysis of the local surface variations yields ±2μm (1σ), which is close to the known production-related surface variations of the coin. To estimate the achievable measurement accuracy, we made an additional measurement for the same star part of the coin. The result is shown in Figure 10b, measured with Keyence confocal microscopy. The analysis shows a measurement accuracy of ±1μm (1σ) over a field of view.

Thus, Figure 7 and Figure 10 provide a comprehensive comparison between speckle measurements using a single LED and the extreme scenario where spatial coherence is distorted by ring illumination. This comparison highlights the inherent differences between the proposed extended aperture ExSPICE profilometry and the standard focus variation technique. It also highlights the need to carefully investigate the trade-offs between the number of LEDs, the spatial coherence of the illumination and the geometric design parameters of both the 4f system and the illumination configuration. It is worth noting that ExASPICE profiling could pave the way for improving both the lateral and axial resolution, thus reducing the measurement accuracy. This observation highlights the potential for further research to clarify the complicated relationships between these parameters and to optimize ExASPICE profiling for specific applications.

## 5. Discussion

We have investigated two cases of the relationship between the numerical apertures of illumination and imaging. By comparing the two scenarios, we also identify inherent differences between the ExASPICE profilometry presented here and the standard focus variation technique.

(i) ${\mathrm{NA}}_{ill}>{\mathrm{NA}}_{img}$: Here, we have managed to reduce the FWHM from 107μm to 27μm, achieving a one-fourth reduction. However, there is a significant decrease in speckle contrast; i.e., the speckle contrast in the image plane disappears due to the incoherent superposition of multiple independent uncorrelated speckle fields. This has a significant impact on the accuracy and reliability of the reconstruction of the 3D height map, which is based on the evaluation of the contrast. In addition, it deprives the ExASPICE profilometry of its advantage of encoding depth information as a function of speckle contrast compared to the focus variation method, which uses surface features such as scratches or dents. Thus, the Michelson contrast (MK) measure cannot be used. However, the fast and non-mechanical depth scanning method distinguishes ExASPICE from the focus variation technique. Experimentally, the 3D shape of the tilted plate from ring illumination measurements, employing the Gaussian derivative (GDER) focus measure in analogy to the focus variation method, was used for reconstruction. The resulting 3D depth map, Figure 7a, displays a height of 342.5μm, differing from the calculated geometrical value by only 0.34μm. However, the surface map shown in Figure 7b and reconstructed from a single LED source, where ${\mathrm{NA}}_{ill}\ll {\mathrm{NA}}_{img}$, reveals additional fine details about the depth of the surface scratches.
(ii) ${\mathrm{NA}}_{ill}\approx {\mathrm{NA}}_{img}$: Here, we achieve a reduction in the FWHM from 62.5μm to 8.8μm, i.e., a factor of eight. We have demonstrated that the redesign of the illumination with the use of a few LEDs has the potential to improve the Michelson contrast evaluation. According to Equation (7), the contrast envelope reaches the minimum value defined by $W_{z,min}$. In this configuration, the speckles generated across the focus plane have maximum contrast while the contrast vanishes across out-of-focus planes. In contrast to the focus variation method, no surface features or textures are required to reconstruct the 3D depth map. The 3D profile of a part from the 2 cent (EUR) coin based on the speckle contrast serves as an example. Here, five sets of intensity measurements are taken with the newly configured illumination. An analysis of the local surface variations yields ±2 μm (1σ), which is close to the known production-related surface variations of the coin. The results demonstrate a twofold improvement in measurement accuracy for ExASPICE compared to SPICE profilometry. Thus, ExASPICE profiling has the potential to enhance the lateral and axial resolution of the measurement.

## 6. Conclusions and Future Works

In this study, we have introduced a new approach to extend the illumination aperture in the recently developed SPICE profilometry technique. The 3D profiler relies on imaging with partially coherent light as previously presented in [11]. The extended illumination aperture is based on the use of multiple LEDs instead of a single LED to illuminate a test object from different directions. We have referred to the new scheme as extended-aperture SPICE, or ExASPICE for short.

As a proof of concept, we conducted experiments to validate the effectiveness of the improved illumination aperture in SPICE profilometry. A significant reduction in the full width at half maximum (FWHM) of the contrast curve was obtained using an equally large numerical aperture (NA) for illumination and imaging; ${\mathrm{NA}}_{ill}\approx {\mathrm{NA}}_{img}$ instead of ${\mathrm{NA}}_{ill}>{\mathrm{NA}}_{img}$. The first case achieves a resolution according to the Rayleigh length of $0.61\lambda /{\mathrm{NA}}^{2}$.

However, further research is needed to carefully investigate the trade-offs related to the number of LEDs, the spatial coherence of the illumination, and the geometric design parameters of both the 4f system and the illumination configuration. This will pave the way for optimizing ExASPICE profilometry for specific applications.

## Data Availability

The data underlying the presented results may be obtained from the authors upon reasonable request.
